# Impact of hyperthermia before and during ischemia reperfusion on neuronal damage and gliosis in the gerbil hippocampus induced by transient cerebral ischemia

**DOI:** 10.1186/cc14543

**Published:** 2015-03-16

**Authors:** CW Park, JH Cho, TG Ohk, MC Shin, MH Won

**Affiliations:** 1 Kangwon National Univeristy, Chuncheon-si, Gangwondo, South Korea

## Introduction

Hyperthermia can exacerbate the brain damage produced by ischemia. In the present study, we investigated effects of hyperthermia before and during ischemia-reperfusion on neuronal damage and glial changes in the gerbil hippocampus following transient cerebral ischemia using cresyl violet staining, NeuN immunohistochemistry and Fluoro-Jade B histofluorescence staining.

## Methods

The animals were randomly assigned to four groups: sham-operated animals with normothermia (normothermia + sham group); ischemia-operated animals with normothermia (normothermia + ischemia group); sham-operated animals with hyperthermia (hyperthermia + sham group); and ischemia-operated animals with hyperthermia (hyperthermia + ischemia group). Hyperthermia (39.5 ± 0.2°C) was induced by exposing the gerbils to a heating pad connected to a rectal thermistor for 30 minutes before and during ischemia-reperfusion.

## Results

In the normothermia + ischemia group, a significant delayed neuronal death was observed in the stratum pyramidale (SP) of the hippocampal CA1 region (CA1) 5 days after ischemia-reperfusion. In the hyperthermia + ischemia group, neuronal death in the SP of the CA1 occurred at 1 day post ischemia, and neuronal death was observed in the SP of the CA2/3 region at 2 days post ischemia. In addition, we examined activation of astrocytes and microglia using immunohistochemistry for anti-glial fibrillary acidic protein (GFAP) and anti-ionized calcium-binding adapter molecule 1 (Iba-1). GFAP-positive astrocytes and Iba-1-positive microglia in the ischemic hippocampus were activated much earlier and much more accelerated in the hyperthermia + ischemia group than those in the normothermia + ischemia group.

## Conclusion

Based on our findings, we suggest that experimentally hyperthermic precondition before cerebral ischemic insult produces more extensive neuronal damage and glial activation in the ischemic hippocampus.

